# cGMP-Dependent Protein Kinase Inhibitors in Health and Disease

**DOI:** 10.3390/ph6020269

**Published:** 2013-02-07

**Authors:** Stefanie Wolfertstetter, Johannes P. Huettner, Jens Schlossmann

**Affiliations:** Department of Pharmacology and Toxicology, Institute of Pharmacy, University Regensburg, Universitätsstr. 31, 93053 Regensburg, Germany; E-Mails: stefanie.wolfertstetter@chemie.uni-regensburg.de (S.W.); johannes.huettner@chemie.uni-regensburg.de (J.H.)

**Keywords:** cGMP, kinase, PKG, inhibitor

## Abstract

cGMP-dependent protein kinases (PKG) exhibit diverse physiological functions in the mammalian system *e.g*., in vascular and gastrointestinal smooth muscles, in platelets, in kidney, in bone growth, nociception and in the central nervous system. Furthermore, PKG were found in insects and in the malaria parasite Plasmodium falciparum. Two different genes of PKG exist: a) the PKG-I gene that is expressed as cytosolic PKG-Iα or PKG-Iβ isoform, and b) the PKG-II gene, which expresses the membrane associated PKG-II protein. The enzyme kinetics, the localization and the substrates of these PKG enzymes differ utilizing different physiological functions. Various inhibitors of PKG were developed directed against diverse functional regions of the kinase. These inhibitors of PKG have been used to analyse the specific functions of these enzymes. The review article will summarize these different inhibitors regarding their specificity and their present applications *in vitro* and *in vivo*. Furthermore, it will be discussed that the distinct inhibition of the PKG enzymes could be used as a valuable pharmacological target e.g., in the treatment of cardiovascular diseases, diarrhea, cancer or malaria.

## 1. Introduction

Kinases are enzymes that transmit phosphate groups from a donor, usually a nucleoside triphosphate (e.g., ATP), to specific substrates. This phosphorylation results in a functional change of the substrate protein. A large group of kinases are protein kinases, which catalyze the transfer to a special amino acid in most cases with a free hydroxyl group.

The most important families are tyrosine kinases and serine/threonine kinases. Examples of serine/threonine kinases are the cGMP-dependent protein kinases (PKG, cGK, respectively) and the cAMP-dependent protein kinase (PKA, cAK, respectively). PKG is mainly activated by the cyclic nucleotide cGMP. In eukaryotes, two different genes were identified: prkg 1 coding for PKG-I and prkg2 coding for PKG-II. By alternative splicing, PKG-I is expressed in two isoforms, PKG-Iα and PKG-Iβ. Both are soluble enzymes, localized in the cytosol. They can interact with different substrates through their individual N-termini. They only differ in their N-terminal domain, the catalytic and the regulatory domains are identical. In contrast PKG-II is bound to the plasma membrane by myristoylation of the N-terminal Gly2 residue. All three PKGs act as homodimers [1,2,3]. Generally, cGMP-dependent protein kinases consist of three domains:

- amino-terminus with a leucine-zipper (important e.g., for homodimerization and targeting) and the autoinhibitory domain.
- regulatory domain with a high- and low-affinity binding site for cGMP (important for the activation of the enzyme).
- catalytic domain for ATP binding, which catalyses the transfer of the phosphate residue to the serine/threonine motif.

PKG-Iα is found in several tissues, mostly lung, cerebellum and heart, whereas PKG-Iβ is expressed predominantly in platelets and hippocampus. Additionally, both kinase isoforms are found in vascular smooth muscle cells, uterus, gastrointestinal tract, kidney and trachea [4,5,6].

The Iα isoform is 10-fold more sensitive to cGMP than PKG-Iβ[7]. This fact results in different activation constants for cGMP: K_a_ (PKG-Iα) = 0.1 µM and K_a_ (PKG-Iβ) = 1.0 µM [8,9,10,11]. PKG-II was detected in kidney, intestine, lung, brain and chondrozytes. Here, the K_a_ for cGMP is 0.07 µM [9,10].

Binding of cGMP releases an inhibition of the catalytic center by the N-terminal autoinhibitory domain and effects the phosphorylation of serine/threonine residues in target proteins. The recognition sequence is K/R K/R X S/T.

Meanwhile, many substrates of PKG-I and PKG-II are identified and well studied. The phosphorylation of these substrates is important for tissue contractility, cell motility, proliferation and differentiation. The substrate specificity depends on the N-terminus of the different isozymes. The isoform PKG-Iα specifically recognizes for example regulatory myosin phosphatase targeting subunit 1 (MYPT1) [12] or RhoA [13]. In contrast the IP_3_RI-associated cGMP kinase substrate (IRAG) is a specific substrate for PKG-Iβ [14]. Cystic fibrosis transmembrane conductance regulator (CFTR) [15] or the transcription factor (SOX9) [16] are specific PKG-II substrates.

Based on the different localization of the kinases the physiological functions are variable. PKG-II regulates the intestinal chloride- and water-secretion [17], is involved in bone ossification [18] and controls renin-release in the kidney [19]. Furthermore PKG-I promotes the opening of calcium-activated potassium channels, which leads to a hyperpolarization and therefore relaxation of smooth muscle cells [20]. A variety of functions of the PKGs are explored in peripheral organs (*e.g*., vasculature, gastrointestinal tract, β-pancreatic cells) and in the CNS (*e.g*., cerebellum, hippocampus).

As mentioned before, the PKGs are the main target for cyclic nucleotides, such as cGMP. This second messenger is synthesized by cyclases (sGC, pGC) after stimulation. The soluble guanylyl cyclase (sGC) is activated by nitric oxide (NO) and carbon monoxide (CO) and effects the conversion of GTP into cGMP. In contrast the particulate guanylyl cylase (pGC) can be stimulated by several natriuretic peptides (CNP, BNP, ANP, *etc*.). The degradation of cGMP takes place by phospho-diesterases (PDEs) via a hydrolysis into 5’-GMP. PDE 5, 6 and 9 are cGMP-specific, while PDE 1, 2, 3, 10 and 11 can convert both cAMP and cGMP [6]. Thus the intracellular concentration of cyclic nucleotides can be controlled. Today several analogs of cyclic nucleotides are known and used as activators or inhibitors for PKG (see below). 

Several reviews exist which describe the diverse activators of PKG [9,21,22]. This review will concentrate on different PKG inhibitors regarding their specificity and their present applications *in vitro* and *in vivo* and a (potential) use in health and disease.

## 2. PKG Inhibitors

### 2.1. Cyclic Nucleotide Analogs

During the last years a variety of cGMP analogs were developed and well described in literature. There are several analogs which act as potent activators (like 8-Br-PET-cGMP) but also as inhibitors for both PKG-I and PKG-II.

**Figure 1:**
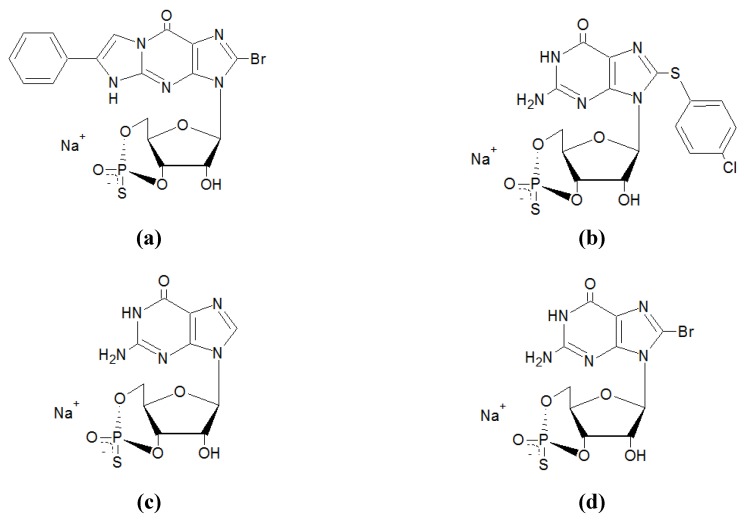
Chemical structures and names of cyclic nucleotide analogs. (**a**) (Rp)-8-Br-PET-cGMP-S, β-phenyl-1,N2-etheno-8-bromoguanosine-3',5'-cyclic monophosphorothioate (Rp- Isomer). (**b**) (Rp)-8-pCPT-cGMP-S, 8-(4-chlorophenylthio)guanosine-3',5'-cyclic monophosphorothioate (Rp- Isomer). (**c**) (Rp)-cGMP-S, Guanosine-3',5'-cyclic mono-phosphorothioate (Rp- Isomer). (**d**) (Rp)-8-Br-cGMP-S, 8-bromoguanosine-3',5'-cyclic monophosphorothioate (Rp- Isomer).

Cyclic nucleotide analogs used as PKG inhibitors are Rp-diastereomers of cGMP. They attach to the cGMP binding domain, so that the enzyme cannot be activated any more, resulting in a competitive, reversible *in vivo* inhibition of PKG-I and PKG-II [22,23]. Furthermore these analogs are membrane-permeable and resistant to hydrolysis by PDEs [24]. Due to their sulphur group in the cyclic phosphate moiety, they are able to inhibit several phosphodiesterases (*e.g*., PDE 5 and PDE 10). The Rp-cGMP-S substances are non-specific antagonists as they inhibit both PKG and PKA [22,25]. (Rp)-8-Br-PET-cGMP-S is the most specific PKG-I inhibitor out of the cyclic nucleotide analogs known until now. (Rp)-cGMP-S also acts as non-specific antagonist and shows a low membrane permeability [22]. Often its structural combination with 8-Br-cGMP is utilized. The resulting substance is (Rp)-8-Br-cGMP-S and is also used as PKG inhibitor. The different structures and the inhibitory constants are shown in Figure 1/Table 1. Due to the low membrane permeability of (Rp)-8-Br-cGMP-S and (Rp)-cGMP-S the *in vivo* use is limited. Meanwhile (Rp)-8-pCPT-cGMP-S and (Rp)-8-Br-PET-cGMP-S are more lipophilic and are able to inhibit PKG in human platelets [26] and intestinal mucosa [27].

**Table 1:** Inhibition constants (K_i_) for the cGMP-dependent protein kinases. The inhibition constants for kinases PKG-Iα, -Iβ and -II and PKA were determined *in vitro.*

Inhibitors	PKG-Iα	PKG-Iβ	PKG-II	PKA-II	Ref.
	K_i_ (µM)	K_i_ (µM)	K_i_ (µM)	K_i _(µM)	
(Rp)-cGMP-S	20	15	0.5	20	[22,25]
(Rp)-8-Br-cGMP-S	3.7	15	-	20	[22]
(Rp)-8-Br-PET-cGMP-S	0.035	0.03	0.45-0.9	11	[3,10,22,24,35]
(Rp)-8-pCPT-cGMP-S	0.5	0.45-0.6	0.29-0.7	8.3	[9,10,22]
KT-5823	0.23	-	-	> 10	[23,28]
H-7	5.8	-	-	3	[1,28]
H-8	0.5	-	-	1.2	[1,28]
H-9	0.9	-	-	1.9	[1,28]
H-89	0.48-0.5	-	-	0.05	[1,23,28]
W45	0.49-1.15	-	-	559	[2,30]
DT-2	0.012	-	-	12.7-20.3	[2,30]
DT-3	0.025	-	-	493	[30]
(D)-DT-2	0.0008	-	-	8.7-15.3	[34]

Abbreviation: - not detected.

### 2.2. K-Series Inhibitors

**Figure 2:**
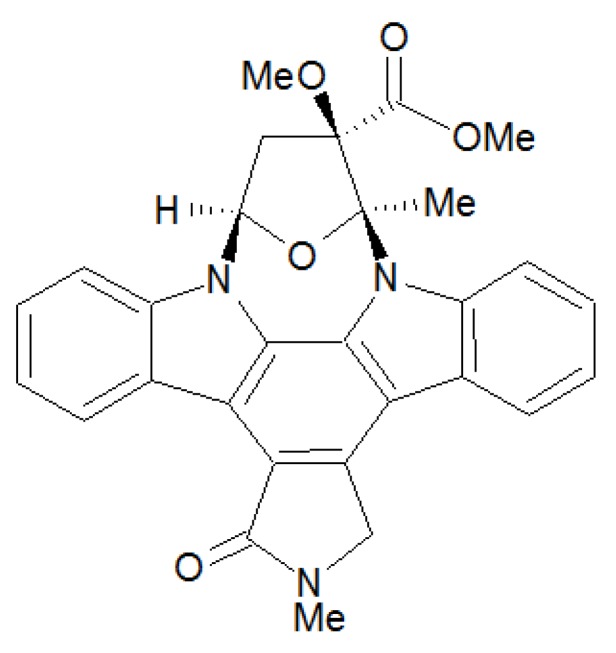
Chemical structure and name of K-Series inhibitor KT5823: (9*S*,10*R*,12*R*)-2, 3,9,10,11,12-hexahydro-10-methoxy-2,9-dimethyl-1-oxo-9,12-epoxy-1*H*-diindolo[1,2,3-*fg*: 3',2',1'-*kl*]pyrrolo[3,4-*i*][1,6]benzodiazocine-10-carboxylic acid, methyl ester.

KT5823 is a potent, *in vitro* inhibitor of PKG, based on the structure of staurosporine (Figure 2). The substance inactivates the ATP-binding site by competition with ATP [23]. KT5823 is an indol carbazole with good membrane permeability. It is also a weak inhibitor of PKC (protein kinase C) and PKA [23,28]. The *in vivo* applicability is not ensured as in cells the inhibitory effect is very low/ cannot be detected [23].

### 2.3. H-Series Inhibitors

**Figure 3:**
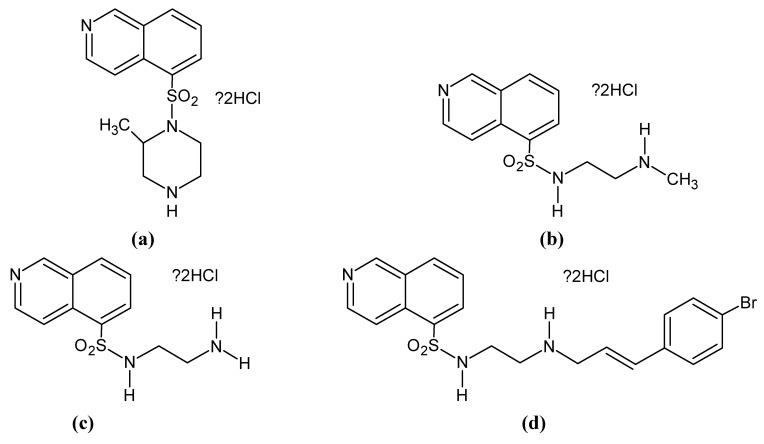
Chemical structures and names of the H-Series inhibitors. (**a**) H-7 hydrochloride, 1-(5-isoquinolinesulfonyl)-2-methylpiperazine·2HCl; (**b**) H-8 hydrochloride, N-[2-(methylamino)ethyl]-5-isoquinolinesulfonamide·2HCl; (**c**) H-9 hydrochloride, N-(2-aminoethyl)-5-isoquinolinesulfonamide; (**d**) H-89, N-[2-(*p*-bromocinnamylamino)ethyl]-5-isoquinolinesulfonamide·2HCl.

The isoquinolinesulfonamide protein kinase inhibitors are also widely used. H-7, H-8, H-9 and H-89 are potent *in vitro* inhibitors of PKG (Figure 3, K_i_ are shown in Table 1). 

The *in vivo* use is discussed [1,28]. H-89 shows very high cell membrane permeability, whereas H-7, H-8 and H-9 can only pass inefficiently. These inhibitors are ATP site inhibitors: via binding at the catalytic ATP sites, they can eliminate the phosphorylation process [23,29]. The inhibitory effect of the H-series substances is not selective: they inhibit PKG, but also PKA, PKC, MLCK (myosin light chain kinase) and diverse other kinases. 

### 2.4. W-Series Inhibitors

**Figure 4:**
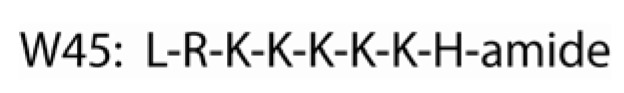
Amino acid sequence (one letter code) of W45

The W-series inhibitors are potent competitive inhibitors for both PKG-I isoforms. They are peptide-based (so they can interact with the substrate domain) and only used *in vitro*, as their membrane permeability is very low [30]. Substances like W45 (Figure 4) are well established and can be taken as control peptide for studies with DT-2 and DT-3, a group of new potent PKG inhibitors (see below). Other W-series inhibitors like W7 and W21 are out of use.

### 2.5. DT Inhibitors

**Figure 5:**
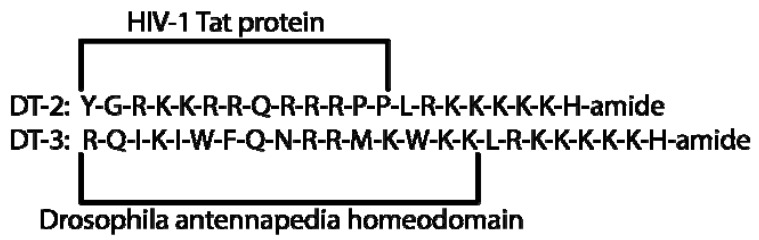
Amino acid sequence (one letter code) and composition of DT-2 and DT-3.

The highly potent DT inhibitor peptides are an improvement of the W-Series inhibitors. These substrate competitive fused-oligopeptides are targeted against the substrate domain and all are membrane-permeant. They do not constrict ATP binding. Via a N-terminal fusion of W45 to the membrane translocation sequences from HIV-1 Tat protein or from Drosophila antennapedia homeodomain the fusion peptide DT-2 or DT-3 are formed, respectively (Figure 5) [30]. DT-2 shows a high selectivity for PKG with a ratio of about 1300 fold compared to PKA [30]. Both PKG and PKA can be inhibited by DT-3, but it is 20000 fold more selective for PKG [30]. The inhibitory constants are shown in Table 1. DT-2 and DT-3 are used for *in vitro* studies; the *in vivo* use is controversial [31,32]. Uptake of DT-2 into cells occurs via endocytic or non-endocytic mechanisms depending on their cellular phenotype [33]. (D)-DT-2 is the D-amino acid analogue of DT-2 and can be used as a potent PKG-Iα inhibitor [34]. This peptide is proteolytically stable and the specificity index (PKG/PKA) can be almost compared to DT-3 with a ratio of approximately 15000 fold [34]. The applicability for *in vitro* studies is ensured; the *in vivo* use is not fully examined yet and depends on the biosystem used [34].

### 2.6. Coccidian PKG Inhibitor

**Figure 6:**
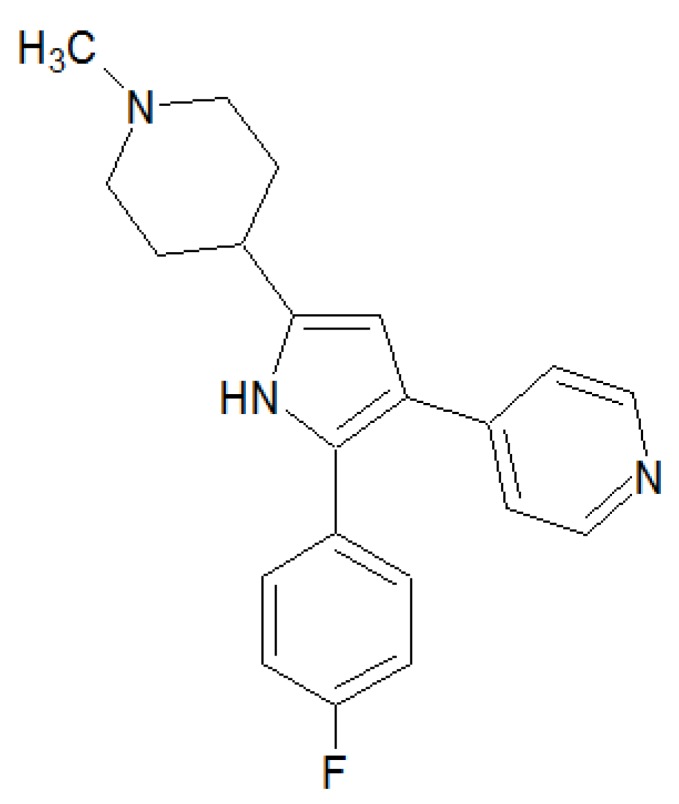
Chemical structure and name of the coccidian PKG inhibitor inhibitor 1, 4-[2-(4-fluorophenyl)-5-(1-methylpiperidine-4-yl)-1*H* pyrrol-3-yl]pyridine.

Inhibitor 1 (Figure 6) inhibits the coccidian PKGs by blocking the ATP-binding site competitively [36]. For more information see 3.4.2.

## 3. PKG-Inhibition as a Potential Therapeutic Target

### 3.1. PKG in Smooth Muscle Organs

#### 3.1.1. PKG in Vascular Relaxation

The influence of PKG-I on vasorelaxation is well established. PKG-I-KO mice show an impaired response to NO/NP induced vasodilatation [37,38,39,40]. Targets of PKG-I that regulate vasorelaxation include inhibition of intracellular Ca^2+^-release from sarcoplasmic/endoplasmic reticulum via IP_3_RI by phosphorylation of IRAG [14]. Ca^2+^-sensitivity of contraction is regulated by an interaction of the PKG-Iα isoform with myosin phosphatase targeting subunit (MYPT) and thereby activation of myosin light chain phosphatase (MLCP) [12,41]. MLCP activation decreases myosin light chain phosphorylation and lead to relaxation with constant [Ca^2+^]. The Ca^2+^-influx through L-type Ca^2+^-channels is indirectly regulated by PKG-I activating large-conductance Ca^2+^-activated maxi-K^+^ channels (BK_Ca_) and thus hyperpolarization of the membrane and closing of voltage-dependent Ca^2+^-channels [39,42]. Due to these mechanisms an increased blood pressure in PKG-I-KO mice was expected. But blood pressure monitoring of those mice showed the expected increase only in juvenile animals whereas adult mice exhibited a normal blood pressure compared to control animals [38]. Under septic conditions due to stimulation with lipopolysaccharides (LPS) IRAG-KO mice did not show the typical hypotonic blood pressure associated with septic shock [43]. The mechanisms described here would favor PKG activators as potential drugs, as a high blood pressure is one of the most common disease in the western hemisphere. Even though an inhibition of PKG would probably lead to severe side effects in prolonged treatment it could be used to antagonize the pathologic hypotonic condition (vasoplegia) encountered during an anaphylactic/septic shock. The efficacy of the PKG inhibitors DT-2 and DT-3 in decreasing NO mediated vasodilation has been demonstrated in isolated cerebral arteries [30,44]. Furthermore investigations on the regulation of PKG-I expression levels revealed a cGMP dependent ubiquitination and degradation of PKG-I in cultured vascular smooth muscle cells. This time- and dose-dependent degradation could be reversed by DT-2 [45].

#### 3.1.2. PKG in Vascular Remodelling

It has been shown in different *in vitro* setups, that NO can have pro- [46,47] and antiproliferative [48,49] effects, depending on the concentration. While most reports describe an antiproliferative effect of PKG on smooth muscle cells, some report the opposite [50], depending on experimental conditions. In a PKG-I knockout, first a pro-proliferative effect was described [51] which was later proven to be a positive effect on cell adhesion [52]. Furthermore dedifferentiated smooth muscle cells have been shown to be a major contributant to the formation of neointimal layers after balloon angioplasty and are also the source of the majority of smooth muscle like cells in atherosclerotic plaques [53,54]. In a smooth muscle specific PKG-I-KO mouse with and without apolipoprotein E-deficient background, an antiproliferative effect as seen *in vitro* could not be reproduced in a restenosis model after carotid ligation [55]. Recent studies with the sGC activator BAY 41-2272 confirm the antiproliferative role of cGMP/PKG in a rat carotid balloon injury model and in cultured VSMC. The antiproliferative effects of BAY 41-2272 could be completely reversed by DT-2 *in vitro* [56]. Once the mechanisms underlying vascular remodelling are fully clarified, PKG inhibitors might become an option to prevent restenosis. Investigations on the effect of DT-2 and DT-3 on angiogenesis in chicken chorioallantoic membranes and rabbit eye cornea identified PKG-I as a downstream effector of vascular endothelial growth factor [57]. These results suggest PKG-I inhibition as a future target in diseases characterized by strong neovascularization. 

#### 3.1.3. PKG in the intestine

While PKG-I is mostly responsible for intestinal motility via the above-explained pathways to decrease smooth muscle tone, studies with the PKG inhibitors KT-5823 and RP-8-pCPT-GMP-S suggest a role in the formation of pacemaker potentials in interstitial cells of Cajal [58]. PKG-II regulates the gastrointestinal secretion of chloride and water through phosphorylation of CFTR [59,60]. This secretion can be stimulated by toxins like the *E. coli* heat stable toxin (STa) to induce diarrhea. STa stimulates the guanylyl cyclase C (GCC) and results via elevation of cGMP and PKG-II activation in phosphorylation of CFTR and thereby trafficking of CFTR from storage vesicles into the apical membrane [15]. This activation and enhanced membrane localization of CFTR results in an elevated Cl^-^ and water secretion. In PKG-II knockout mice STa does not induce this diarrhea [61], clearly marking PKG-II inhibition as a potential target to treat toxin-induced diarrhea.

### 3.2. PKG in the Bone

#### 3.2.1. PKG in Bone Development

During normal bone development chondrocytes arise from a mesenchymal colony, undergo a proliferative state and finally differentiate into hypertrophic chondrocytes that express cartilage matrix. In a last step this cartilage matrix is calcified and replaced by bone. The naturally occurring Komeda miniature rat Ishikawa (KMI) contains a deletion in the Prkg2 gene resulting in a frame shift and a premature stop codon in the transcript. Thereby, a truncated PKG-II is expressed that lacks the kinase domain [62]. In these rats the switching from proliferative chondrocytes to hypertrophic chondrocytes is impaired and results in a diminished longitudinal growth of bones. The involvement of PKG-II in bone development and growth was also shown in PKG-II-KO mice [61]. Recent studies have identified glycogen synthase kinase 3β (GSK-3β) as a likely substrate for PKG-II that mediates the kinases influence on skeletal growth through hypertrophic differentiation of growth plate chondrocytes [63]. Furthermore, a function for PKG-II in osteoblast mechanotransduction and in the osteoblast anabolic response via extracellular signal-regulated kinases (ERK), sarcoma tyrosine kinase (SRC) or transcription factor c-fos was proposed [64,65]. These results point to a teratogenic potential of PKG inhibitors if given during pregnancy. Investigations of the PKG-inhibitor DT-3 and siRNA knockdown of PKG-Iα on the bone marrow stromal cell line OP-9 suggest a role of PKG-Iα in bone marrow functionality [66]. As bone marrow stromal cells provide a micro-environment for hematopoietic stem cells, treatment with PKG inhibitors might ultimately lead to bone marrow failure.

### 3.3. PKG Signaling in Cancer

#### 3.3.1. PKG-Iα in Lung Cancer

It is known that PKG-Iα signaling has cytoprotective and anti-apoptotic effects in various issues [50,67] as far as conferring chemoresistance in ovarian cancer cells [68]. Investigations into the PKG-Iα cytoprotective effects in non-small-cell lung carcinoma cell lines H460 and A549 demonstrated, that specific inhibition of PKG-Iα with the inhibitor DT-2 significantly increases spontaneous apoptosis. SiRNA-mediated knockdown of PKG-Iα led to a reduced expression of the inhibitor of apoptosis proteins c-IAP1 (cellular inhibitor of apoptosis-1), livin and survivin. Treatment of the cisplatin resistant cell line A549 with a combination of cisplatin and DT-2 showed a synergistic effect in induction of apoptosis whereas pretreatment of A549 cells with 8-Br-cGMP caused significant protection towards cisplatin [69]. These results mark PKG-Iα inhibitors as a potential co-medication in cisplatin chemotherapy of solid tumors.

#### 3.3.2. PKG in Colorectal Carcinoma

It was recently shown that NO/PKG/extracellular-signal-regulated kinases (ERK) signaling promoted migration and invasion of colorectal carcinoma (CRC) cell lines in both scratch wound and modified Boyden chamber assays [70]. Treatment with the NO-donor *S*-nitroso-*N*-acetylpenicillamine (SNAP) led to stronger migration whereas co-treatment with SNAP and the GC-inhibitor 1*H*-[1,2,4]oxadiazolo[4,3-a]quinoxalin-1-one (ODQ), the PKG-inhibitor KT5823 or the ERK-inhibitor PD98059 reversed this effect. Further investigations showed that SNAP treatment led to an up regulation on mRNA level as well as an activation of RhoGTPases and matrix metalloproteinases (MMPs) that were also reversible with the aforementioned inhibitors.

These results offer another potential target for PKG inhibitors during the treatment of early stages of CRC to reduce the risk of metastasis formation. However, proapoptotic effects of PKG were reported in the analysis of the PKG inhibitor DT-2 in the colon epithelial cell line CCD841 and the transfection of SW480 and SW620 colon carcinoma cell lines with PKG [71].

#### 3.3.3. PKG in Breast Cancer

In contrast to the rather positive effects on cancer mentioned so far, recent reports imply a pro apoptotic role of PKG in an estrogen receptor-positive (MCF-7) and –negative (MDA-MB-468) breast cancer cell lines [72]. Treatment of these cell lines with 1-benzyl-3-(5’-hydroxymethyl-2’-furyl)indazole (YC-1), a sGC activator, and 8-Br-cGMP in varying concentrations inhibited the viability in a time and dose dependent manner. FACS-analysis with an Annexin-V/propidium iodide stain of treated cells showed an increase in the apoptotic cell fraction. These apoptotic effects could be reversed by co-treatment of the cells with one of the activators and either KT5823 or Rp-8-pCPT-cGMP-S. The results presented in this chapter place PKG inhibition in cancer treatment in an ambiguous role. In some forms of cancer the effects seem to be very positive where in other forms a rather negative effect was demonstrated. To utilize PKG inhibition in cancer treatment, a minute classification of the malignancy and its response to the treatment options is vital. 

### 3.4. PKG Provides a Target for Parasite Treatment

#### 3.4.1. Interspecies Differences in PKG

**Figure 7:**
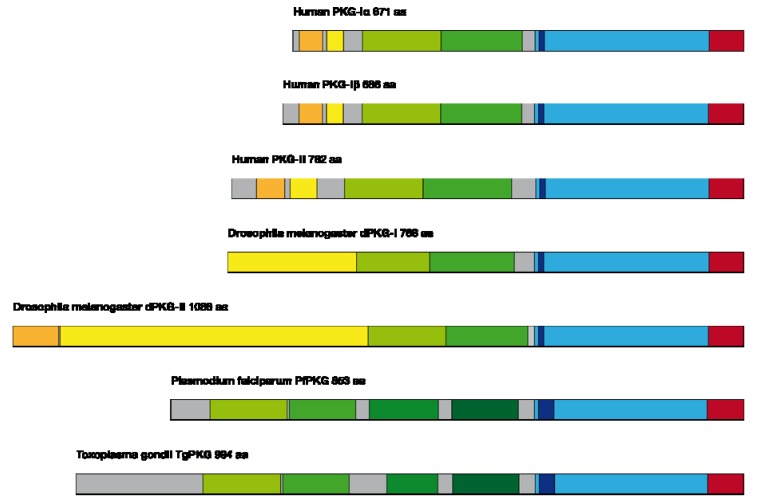
PKG enzymes in different species light blue=kinase domain, dark blue=ATP-binding site, green=cGMP-binding sites 1-4, yellow=regulatory domains, orange=interaction domains, red=C-terminal AGC (cAMP-dependent, cGMP-dependent and protein kinase C)-kinase domain.

As shown in Figure 7 PKG proteins vary largely between species. Major variations can be found in length, from the relatively short 671 aa enzyme PKG-I in mammals to the large 1088 aa dPKG-II isozyme in Drosophila melanogaster, in cGMP binding sites 2–4 and in regulatory and interaction domains. These differences offer potential targets for the development of species specific drugs that can be utilized e.g., in parasite treatment.

#### 3.4.2. PKG: A Novel Target in Malaria Therapy

It has been shown that the coccidian PKGs are a target for the inhibitor 4-[2-(4-fluorophenyl)-5-(1-methylpiperidine-4-yl)-1*H* pyrrol-3-yl]pyridine (inhibitor 1) [36]. The inhibitor competitively blocks the ATP binding site and does not influence mammalian PKG. Experiments with *Plasmodium falciparum s*chizonts suggest a role of *Plasmodium falciparum* PKG (PfPKG) in schizont progression and rupture. Specifity of inhibitor 1 for PfPKG was demonstrated by treatment of an inhibitor-insensitive transgenic parasite [73]. PfPKG expression is upregulated during the schizont stages and reaches its peak in the segmented schizont stage [74].

Inhibitor 1 was also tested in *T. gondii* and *E. tenella* and insensitive transgenic parasites to establish a role of PKG in the release of adhesive proteins, motility, attachment and invasion of host cells [75]. A potential problem of malaria therapy with coccidian PKG inhibitors is the development of resistances. The inhibitor-insensitive transgenic parasites used to demonstrate PKG specifity of the effect differed only in a single amino acid in the ATP-pocket of the enzyme. To utilize PKG as a clinical target, more reliable inhibitors have to be developed.

**Figure 8:**
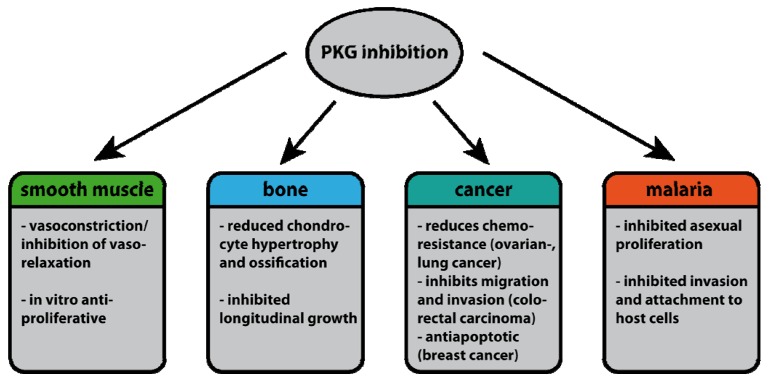
Effects of PKG inhibition on various targets.

## 4. Conclusions

In this review the different available PKG inhibitors were summarized and the effect of PKG inhibition in various organ systems was presented. Current inhibitors of PKG activity target the ATP-binding, the cGMP-binding or the substrate recognition site. Some of these inhibitors (e.g., of the W-Series or DT-series) are highly specific compared to its inhibition of PKA. Diverse inhibitors can be used for analytical aspects *in vitro*. However, the application of these inhibitors *in vivo* is still tempting. Hence, there is a further need for the development of more specific and *in vivo* usable inhibitors to enhance its applicability *in vitro* and *in vivo*. The different physiological functions of PKG imply that its inhibition will yield in various pleiotropic effects (Figure 8). PKG-I inhibition in smooth muscle organs reduces vasorelaxation, influences vascular angiogenesis and retards intestinal motility. PKG-II inhibition in the intestine prevents toxin-induced diarrhea. PKG-II is also involved in bone development. In various cancer cells PKG inhibition suppresses proliferation and might influence apoptosis. PfPKG inhibitors could be useful in the treatment of various stages of malaria. Therefore, studies of PKG inhibitors might lead to new directions regarding therapeutic applications. Furthermore, they might guide to explanations of side effects of cGMP-enhancing substances or possible PKG activators. 
